# A framework to analyze opinion formation models

**DOI:** 10.1038/s41598-022-17348-z

**Published:** 2022-08-04

**Authors:** Carlos Andres Devia, Giulia Giordano

**Affiliations:** 1 Delft Center for Systems and Control, Delft University of Technology, 2628 CN Delft, The Netherlands; 2 Department of Industrial Engineering, University of Trento, 38123 Trento, Italy

**Keywords:** Applied mathematics, Computational science

## Abstract

Comparing model predictions with real data is crucial to improve and validate a model. For opinion formation models, validation based on real data is uncommon and difficult to obtain, also due to the lack of systematic approaches for a meaningful comparison. We introduce a framework to assess opinion formation models, which can be used to determine the qualitative outcomes that an opinion formation model can produce, and compare model predictions with real data. The proposed approach relies on a histogram-based classification algorithm, and on transition tables. The algorithm classifies an opinion distribution as *perfect consensus*, *consensus*, *polarization*, *clustering*, or *dissensus*; these qualitative categories were identified from World Values Survey data. The transition tables capture the qualitative evolution of the opinion distribution between an initial and a final time. We compute the real transition tables based on World Values Survey data from different years, as well as the predicted transition tables produced by the French-DeGroot, Weighted-Median, Bounded Confidence, and Quantum Game models, and we compare them. Our results provide insight into the evolution of real-life opinions and highlight key directions to improve opinion formation models.

## Introduction

During the past 30 years, the study of opinion formation has attracted growing attention^1–7^. An *opinion formation model* is a mathematical model aimed at reproducing the evolution of opinions within a population in a given time interval. Several opinion formation models have been proposed, where opinions can be continuous^8,9^ or discrete^10,11^ variables, and can evolve in discrete^12,13^ or in continuous^14,15^ time in a deterministic^13^ or stochastic^16^ way, over an underlying interaction graph that can be time-varying^17–19^, directed, weighted, or signed^20^. Opinions can be uni- or multi-dimensional^21,22^. When studying the behaviors emerging from these models, the focus is not on individual opinions, but on the overall evolution of opinions in the entire population. Denoting as *opinion distribution* the collection of all the opinions within a population at a given time instant, opinion formation models address two main questions: (i) *given a set of parameters and an initial opinion distribution, what will be the opinion distribution after some time?* and (ii) *under which circumstances will a desired opinion distribution be achieved?* The answers depend on the chosen model. For instance, the French-DeGroot model is guaranteed to asymptotically achieve *perfect consensus* (all individuals share the very same opinion) if the graph is strongly connected^2^. For a structurally balanced digraph, the Altafini model predicts *polarization* (presence of two opposed opinion groups) if the digraph is strongly connected^20,23^ and *consensus* (all individuals have almost the same opinion) near the origin, if it has a spanning tree^24,25^. When bounded confidence is added to the model, then *clustering* (presence of several distinct opinion groups) is a likely outcome^26^. In addition, we call *dissensus* a practically uniform distribution of the opinions.

We identified *perfect consensus*, *consensus*, *polarization*, *clustering* and *dissensus* as qualitative categories of opinion distributions that emerge in real life. They recurrently appear in the results of the World Values Survey^27–29^, conducting global surveys every 5 years; in particular, we monitored the answers to 30 questions (regarding values, behavior, and ethics) asked to participants in 25 countries in three occasions separated by roughly 5 years, corresponding approximately to the years 2010 (wave 5), 2015 (wave 6), and 2020 (wave 7).

A complete opinion formation model should be able to produce, with the appropriate parameter choice, each of the qualitative opinion distribution categories found in actual societies, as well as each of the possible transitions, from one category to the others, that occur in reality. Here, we introduce a framework to systematically check whether this is the case.

First, we introduce a *histogram-based classification algorithm* to associate an opinion distribution with a qualitative category. Histogram-based classification has been used in many fields, especially related to image processing^30,31^; yet, to the best of our knowledge, this is the first time it is adopted in an opinion-dynamics setting. Second, we construct a *transition table* to visualize how opinion distributions evolve over time from an *initial* to a possibly different *final* qualitative category.

The proposed framework (which leverages histogram-based classification to associate opinion distributions with qualitative categories, and transition tables to capture the evolution of opinions over time) allows to assess and analyze opinion formation models and compare their prediction with real data. We demonstrate our novel approach by applying it to models already proposed in the literature. In particular, we compare real-world data, representing opinion distributions at different sampling times (waves) taken from the World Values Survey^27–29^, with the predictions provided by various well-known opinion formation models: French-DeGroot^12,13^, Weighted Median^32^, Bounded Confidence^33–35^, and Quantum Game^36^ models.

An extensive literature deals with model comparison based on Bayesian analysis: Bayes factor^37–39^, Bayesian evidence^40^, and Bayesian methods such as the $L_v$ measure^41^. Model comparison statistics^42^ can be based on the information criterion^43^ or the deviance information criterion^44^. These statistical model comparisons aim at selecting the model that fits at best a given data set. Conversely, simulation-based model comparison directly compares the outcomes of two or more models without fitting real data^45,46^. As a main novelty, we propose a peculiar simulation-based framework that compares opinion formation models not in their ability to reproduce a given data set, but in their capability to generate *a spectrum of qualitative behaviours that is as broad as the one observed in real life*.

Our results provide insight into real-life opinion evolution and comparatively assess different opinion formation models. They reveal that, while *all* transition between qualitative categories occur in reality, existing models can only yield *some* peculiar transitions and are characterized by a *bias towards consensus* that cannot be found in real opinion data.

The paper is structured as follows. First, we introduce the proposed framework to analyze opinion formation models and compare their predictions with real data: we describe our approach to classify opinion distributions and our framework allowing a systematic comparison between model predictions and real-life opinions. Then, we showcase examples of application of the proposed approach to well-studied existing opinion formation models, whose predictions are compared with real-life opinion data from the World Values Survey: we show that real transition tables highlight some characteristic features of opinion evolution in real life, and we compare the predicted transition tables with the real ones for different opinion formation models, including both classical and quantum models.

## Results

**Figure 1:**
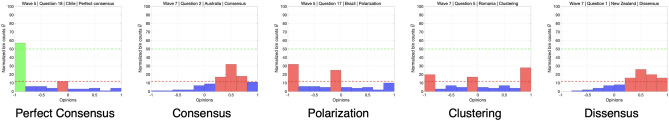
Examples of real-life opinion distribution histograms, taken from the World Values Survey data, classified as perfect consensus, consensus, polarization, clustering, and dissensus by the proposed *histogram-based classification algorithm*. The vertical axis represents the normalized bin counts $\tilde{H}$. The dotted lines mark thresholds $T_1$ (green) and $T_2$ (red). The bins are colored according to the histogram classification algorithm: green if the normalized bin count is larger than $T_1$; blue if it is smaller than $T_2$; red if it is between $T_1$ and $T_2$. The parameter values we adopted to perform the classification are: $M=10$, $B=3$, $K=3$, $T_1=50$, and $T_2=12$.

We denote as *opinion* the level of agreement with a statement. The opinions that an individual can have belong to the interval $[-1,1]$, where the values $-1$, 0, and 1 respectively denote complete disagreement, indifference, and complete agreement with the statement. Given a population of $N$ individuals, each having an opinion about a statement, the collection of the opinions of all the individuals in the population yields an *opinion distribution*, which belongs to one of our identified qualitative categories, exemplified in Fig. 1: perfect consensus, consensus, polarization, clustering, and dissensus. Their mathematical definitions, provided in the following, are inspired by these informal definitions (where by absolute majority we denote more than 50% of the population):

- *Perfect consensus* the absolute majority chooses the very same opinion;
- *Consensus* the absolute majority chooses approximately the same opinion;
- *Polarization* the absolute majority is split between two ‘distant’ opinions;
- *Clustering* the absolute majority is split between two or more groups;
- *Dissensus* the majority of the opinions are uniformly distributed.

These categories of opinion distributions capture an increasing level of inhomogeneity. When all the individuals have the exact same opinion (perfect consensus), there is perfect homogeneity. Starting from perfect consensus, progressively increasing inhomogeneity leads to consensus, polarization, clustering, and lastly dissensus. When every opinion is held by the same number of people (perfect dissensus), inhomogeneity is maximal and no preference whatsoever can be identified.

### Opinion distribution classification

An opinion distribution can be visualized as a histogram with $M$ bins, which can then be classified so as to determine to which qualitative category the opinion distribution belongs. This process is performed by our proposed *histogram-based classification algorithm*.

1. Input the positive integers $M$, $B<M$ and $K\le M-2$, and the thresholds $T_1$, $T_2$ with $0<T_2<50 \le T_1<100$.
2. Partition the $[-1,1]$ interval in $M$ bins of equal width.
3. Count how many opinions fall in each bin. Denote by *H*(*k*) the number of opinions in bin *k* ($1 \le k \le M$).
4. Normalize the bin counts so they add up to 100. Denote the normalized bin counts by $\tilde{H}(k)$.
5. Classify each bin as *green*, *blue*, or *red*: bin *k* is green if $\tilde{H}(k)>T_1$; blue if $\tilde{H}(k)<T_2$; red otherwise.
6. Compute the *number of groups*; a group is formed by consecutive green or red bins. For each group, compute the *number of bins*, and the *normalized group count*, which is the sum of all the normalized bin counts of the bins belonging to the group.
7. Classify the histogram according to the following criteria:
  *Perfect consensus* if there is a green bin;
  *consensus* if there is one group, with at most $B$ bins, and with normalized group count larger than 50;
  *Polarization* if there are two groups, each with at most $B$ bins, with at least $K$ bins in between, whose normalized group counts add up to more than 50;
  *Clustering* if there are two or more groups, each with at most $B$ bins, whose normalized group counts add up to more than 50;
  *Dissensus* otherwise.

The parameters $M$, $B$, $K$, $T_1$, and $T_2$ allow the proposed classification to be tuned according to the problem at hand, thus taking into account possible differences in the interpretation of our proposed qualitative categories.

In our case study, $M= 10$ is a natural choice, since the data from the World Values Survey^27–29^ comes from Likert 10-scale questions. Parameter $B$ represents the ‘level of closeness’ required to state that a group of people share a ‘similar’ opinion: we set $B=3$. Polarization is defined as the presence of two groups with significantly opposing views; the required ‘level of opposition’ is encoded by the parameter $K$. Two groups at a distance less than $K$ would represent clustering, since the opinions are not very different, while two groups at a distance $K$ or more represent two significantly opposing views, and hence polarization. The value $K= M- 2$ would mean that extreme opposition is needed to define polarization; in this paper, we choose $K=3$. The threshold $T_1$ defines perfect consensus: we choose $T_1=50$ to capture all instances where the absolute majority (more than 50%) shares a single opinion. The threshold $T_2$ discriminates between significantly numerous opinion groups and ‘white noise’ residual opinions. A low $T_2$ leads to the appearance of multiple groups with more than *B* bins, while a high $T_2$ leads to interpreting significant opinion groups as white noise: in both cases, the classification is biased towards dissensus. After repeated numerical experiments, the intermediate value of $T_2 = 12$ was selected and can be seen as a robust choice, because varying $T_2$ between 10 and 14 gave comparable classification results.

Figure 1 shows examples of real-life opinion distribution histograms, taken from the World Values Survey data, representative of our proposed qualitative categories.

### Model predictions versus real opinions: a framework for systematic comparison

The proposed *histogram-based classification* approach allows us to systematically associate a given opinion distribution, which can be either real (e.g. survey data) or predicted by an opinion formation model, with a qualitative category. An opinion distribution is a static snapshot; to study opinion formation, we need to understand how opinion distributions can evolve over time. We introduce *transition tables* to capture the possible qualitative categories of final opinion distribution that can be obtained, after some time, starting from various categories of initial opinion distribution. A transition table is a matrix whose rows (respectively, columns) are associated with the qualitative category of initial (respectively, final) opinion distribution: entry (*i*, *j*) represents the number of opinion distributions that evolve from an initial configuration belonging to category *i* to a final configuration belonging to category *j*, where *i* and *j* can be either perfect consensus, consensus, polarization, clustering, or dissensus. To systematically compare the outcome of a given opinion formation model with real opinion data collected at two different time instants, we proceed as follows:

1. Classify the *real* initial opinions,
2. Let them evolve according to the opinion formation model, and produce the *predicted* final opinions,
3. Classify the *predicted* final opinions,
4. Using the classification of *real* initial opinions and *predicted* final opinions, construct the *predicted* transition table,
5. Classify the *real* final opinions,
6. Using the classification of *real* initial opinions and *real* final opinions, construct the *real* transition table, and
7. Compare the two transition tables.

**Figure 2:**
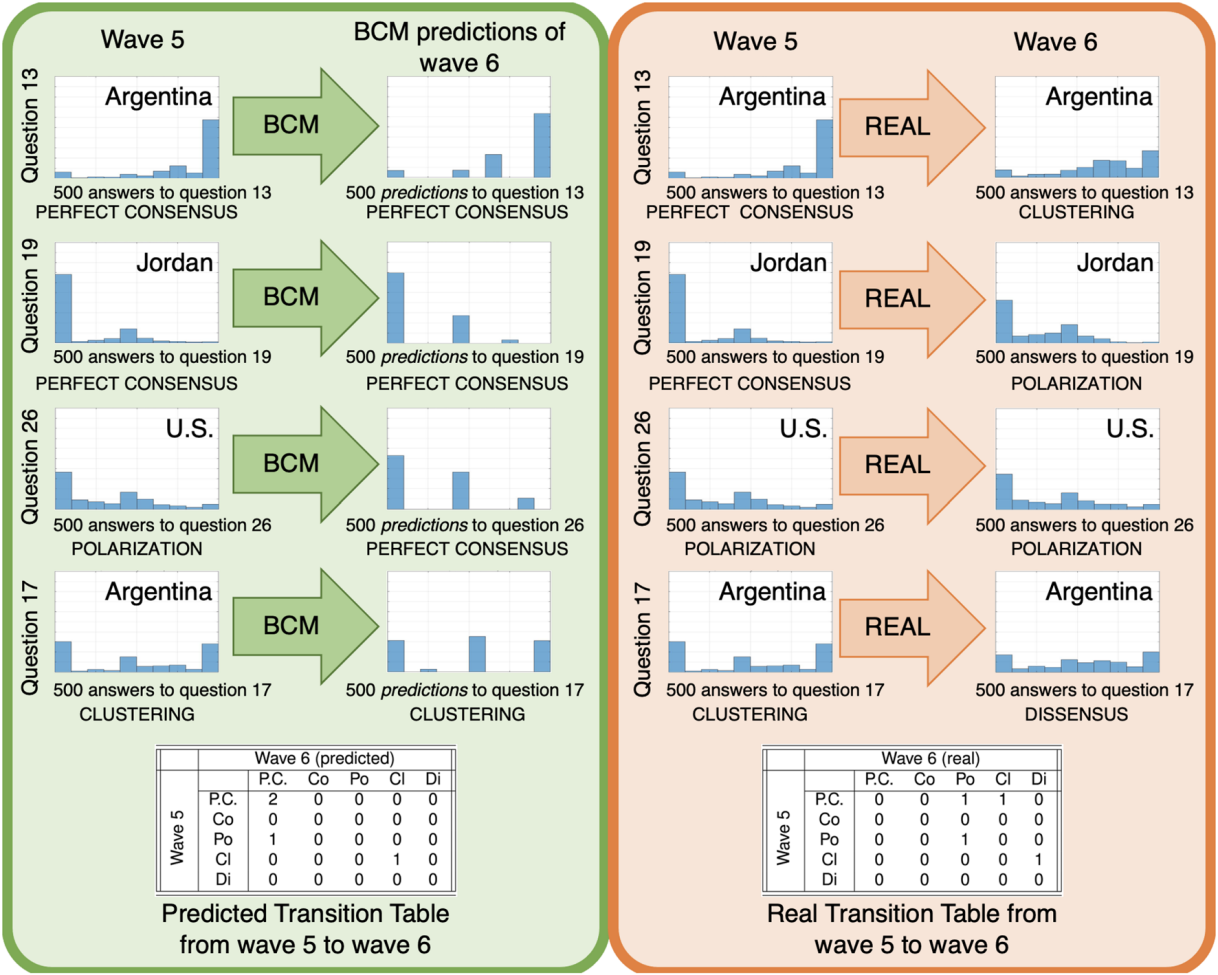
Simple example to illustrate the proposed approach: based on the answers to 4 questions administered to 500 people in two consecutive survey waves, the accuracy of the Bounded Confidence Model (BCM) can be assessed by comparing the *predicted* transition table (left) with the *real* transition table (right). In the tables P.C. is perfect consensus, Co is consensus, Po is polarization, Cl is clustering, and Di is dissensus. This example is simply aimed at showcasing how the approach works, so any model could have been chosen; the BCM was selected because it is a widely used, well-known and well-studied model.

As an example, we assess the Bounded Confidence model (BCM)^8,9,33–35^, with confidence radius 0.3, along with the answers, provided by 500 people, to four questions of the World Values Survey both in wave 5 (2010) and wave 6 (2015). The four initial opinion distributions (wave 5) are classified by our algorithm as perfect consensus, perfect consensus, polarization, and clustering, respectively. Taking these opinion distributions as initial conditions, the Bounded Confidence model yields *predicted* opinion distributions that our algorithm respectively classifies as perfect consensus, perfect consensus, perfect consensus, and clustering. Hence, two opinion distributions are predicted to remain perfect consensus, one to change from polarization to perfect consensus, and one to remain clustering, as summarized in the *predicted* transition table in Fig. 2 (left). The *real* transition table in Fig. 2 (right) can be constructed by considering the *real* final opinion distributions (wave 6) for the same four questions, which are classified as clustering, polarization, polarization, and dissensus, respectively.

Comparing real and predicted transition tables allows us to evaluate the model, identify its shortcomings and suggest ways to improve its realism. Furthermore, real transition tables provide qualitative understanding of how the actual opinion distributions can evolve within the population in the considered time interval.

### Real transition tables highlight features of opinion evolution

**Figure 3:**
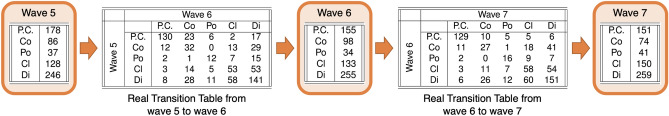
Real transition tables: The 675 real opinion distributions emerging from the World Values Survey^27–29^ waves 5, 6, and 7 are qualitatively classified as perfect consensus (P.C.), consensus (Co), polarization (Po), clustering (Cl), and dissensus (Di) in the orange panels. The transition tables show the qualitative evolution of opinion distributions between these waves, highlighting how each qualitative category of opinion distributions could evolve into the various other categories.

We analyzed in total 2025 real opinion distributions, corresponding to World Values Survey answers to 30 questions in 25 countries in waves 5, 6, and 7, approximately corresponding to years 2010, 2015, and 2020^27–29^; not all questions were asked in all countries, hence there are 675 opinion distributions for each wave. The orange panels in Fig. 3 show the qualitative classification of all the opinion distributions in each wave. The number of opinion distributions belonging to each qualitative category does not change significantly in different waves and a recurrent pattern can be observed: dissensus is consistently the most common outcome, followed by perfect consensus and then by clustering, by consensus and finally by polarization, which is invariably the least common outcome. Figure 3 also reports the *real* transition tables from wave 5 to 6, and 6 to 7, which evidence that, in spite of the observed recurring pattern, opinion distributions themselves do not tend to remain in the same category. On the contrary, there are several examples of opinion distributions that move from a category to almost any of the others: the real transition tables indicate that, in real life, opinion distributions can evolve from any category to any other. The likelihood of evolving towards a different qualitative category can be assessed by comparing the sum of diagonal and off-diagonal entries in the transition tables: from wave 5 to wave 6, these numbers are 368 and 307 respectively, indicating that around $45\%$ of the opinion distributions move to a different qualitative category; from wave 6 to wave 7, these numbers are 381 and 294 respectively, hence the probability of change has decreased to roughly $44\%$. Interesting similarities emerge in the patterns of the two transition tables: corresponding entries often have close values, or at least the same order of magnitude, which seems to suggest that the likelihood of evolving from a qualitative category to another changes slowly over time.

### Predicted transition tables and model comparison

**Figure 4:**
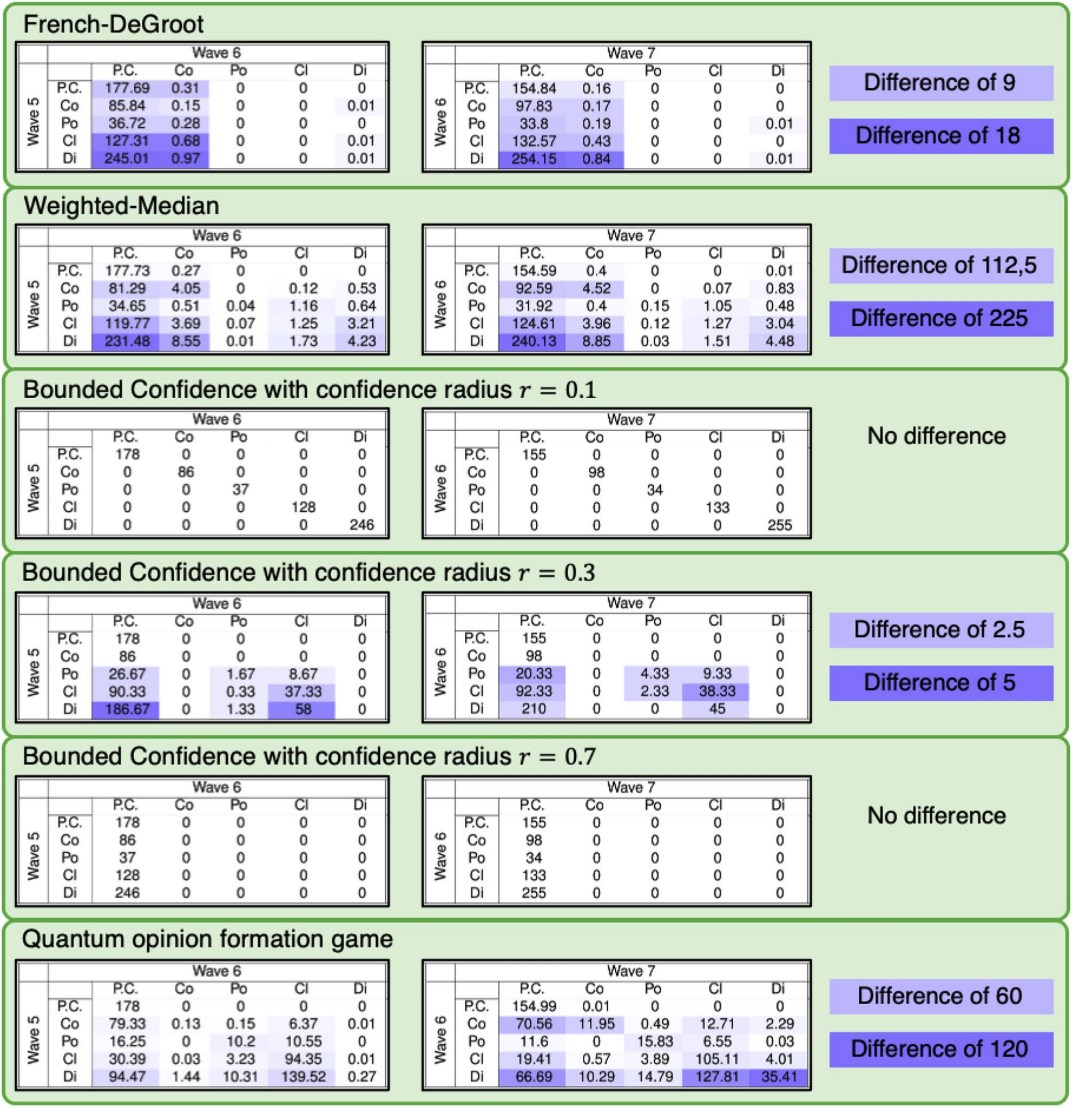
Average predicted transition tables from wave 5 to 6 (left), and 6 to 7 (right) for the six considered models. Each table entry contains the average of the corresponding values in the 75 computed transition tables, obtained for different population sizes, graph topologies, and initial opinion assignments. The variability of these values, in terms of the difference between the maximum and minimum value across all 75 tables, is denoted by the shade of blue. A cell with light blue color  represents half the maximum difference for that model, and a cell with dark blue color  represents the maximum difference for the model. The value of the difference represented by these shades of blue is reported to the right. A white cell means that all values are identical for all 75 tables.

Starting from the opinion distributions in wave 5 and wave 6, we generated the next wave results predicted by six different opinion formation models: French-DeGroot (FG), Weighted-Median (WM), Bounded Confidence with confidence radius 0.1 (BC1), 0.3 (BC2), and 0.7 (BC3), and Quantum Game (QG). Figure 4 shows the obtained *average* predicted transition tables, from wave 5 to 6 (left) and from wave 6 to 7 (right): each reported transition table is the average over 75 tables, computed for different population size, directed graph topology, and initial opinion assignment (details are in the Materials and Methods section and Supplementary Information). The shade of blue quantifies the variability across the 75 tables represented by the difference between the maximum and minimum value for each cell across all 75 transition tables. The exact variability value for each cell in the transition tables for all the methods can be found in Tables 10 and 11 of the Supplementary Information.

The French-DeGroot (FG) model behaves as expected, with a clear trend towards perfect consensus, evidenced not only by the average transitions but also by the low difference. However, it is interesting to note that not all final opinions result in perfect consensus, some produce consensus, and in exceptional circumstances dissensus. There are two explanations for these cases. First, in large interaction graphs it takes more time to achieve perfect consensus because, even if the graph is strongly connected, only few edges may be responsible for that strong connectivity, thus the graph could have two or more ‘pseudo clusters’. The second reason is that, if the opinion towards which agents converge is in the middle of two histogram bins, it may happen that the two adjacent bins to that converging opinion are equally populated, thus resulting in consensus and not perfect consensus.

The Bounded Confidence models with confidence radius $r=0.1$ (BC1) and $r=0.7$ (BC3) also behave as expected: for the BC1 model, the confidence radius is so small that most of the edges vanish in the first step and then the opinions remain the same, hence the transition table is a diagonal matrix. On the other hand, the BC3 model produces exclusively perfect consensus. The reason why BC3 produces perfect consensus always, while the same does not happen with FG, is that BC3 creates more edges in the interaction graph. Hence it is possible to assume that after a few time steps the interaction graph is complete, and then the convergence to a single opinion is much faster, resulting in perfect consensus. It is also interesting to note that BC1 and BC3 are the models showing the smallest variability (difference of zero).

The Bounded Confidence model with an intermediate value of confidence radius $r=0.3$ (BC2) is biased towards perfect consensus, but allows some instances of polarization, clustering and dissensus to evolve into polarization or clustering. A larger confidence radius (with respect to BC1) yields strongly connected subgroups of individuals that achieve perfect consensus among them: if there are only two subgroups with sufficiently distant opinions, polarization occurs, otherwise the model produces clustering, which is the most likely outcome of the two. No consensus outcomes are generated, because, once the opinions are sufficiently close, they evolve into perfect consensus. For this model, varying population size, graph topology, and initial opinion assignment appears to have little impact, as seen in a maximum difference of 5.

The Weighted Median (WM) model exhibits a very rich behaviour. Although biased towards perfect consensus, it can produce every transition except the ones from perfect consensus and consensus to polarization, and from perfect consensus to clustering. This wide range of outcomes is accompanied by a high sensitivity with respect to varying population size, graph topology, and initial opinion assignment, which is the highest across all considered models. The bias towards perfect consensus is expected, given the fact that the WM model is based on the cognitive dissonance theory and conformist tendencies.

Finally, the Quantum Game (QG) model presents a very interesting transition table. There is a tendency towards consensus, which is consistent with the fact that agents can only *Change*, *Keep*, and *Agree*, hence there is no disagreement mechanism. However, the randomness with which agents are chosen to interact, along with the dependence of the payoff matrices on the opinion distance, also produces a clustering behaviour (a bounded confidence effect). Therefore, when the initial distribution is perfect consensus, most agents will interact with each other, but the final opinion will be almost the same, resulting in perfect consensus; when the initial distribution is consensus, then there is a tendency to perfect consensus, but the agents that are not in the consensus bins can move other agents away, resulting in some clustering; when the initial distribution is polarization, there is a greater chance of producing polarization or clustering, due to the bounded confidence effect, and this pattern is also present when the initial conditions correspond to clustering or dissensus. Another interesting observation is that the QG model can evolve with a lower ‘speed of change’. The facts that only two agents are chosen to interact at each time step and that they may not change opinion creates the possibility that, in a considerable fraction of time steps, the opinions do not change, in contrast with the other ‘classic’ opinion formation models, where the opinions are constantly changing.

## Discussion

Several opinion formation models have been proposed in the literature, often based on well-studied sociological and psychological principles, such as social conformity theory^47,48^, credibility^49^, biases^50–52^, trust^53,54^, strong and weak ties^55,56^, moral foundations^57^, expertise^58^, stimulus-response theory^59^, stimulus-object-response theory^60^, ‘back-fire’ effect^61,62^ and ‘boomerang’ effect^63^, among many others. Significant effort has been devoted to analysing opinion formation models, but their predictions are rarely compared with real data. A notable exception is the Friedkin-Johnsen model, which has been validated on numerous experiments with *small* and *medium*-size groups^6,7,22,64,65^. However, comparing the model results with *large scale data* is a challenging task for several reasons, including the difficulty in collecting large amounts of reliable opinion distributions at subsequent time instants and in gathering information about the topology of the corresponding interaction network^7^, as well as the lack of systematic approaches for a high-level comparison between qualitative model outcomes and real data. Despite the difficulties, comparison with real data is crucial to assess the usefulness of a model and to identify possible directions for improving it.

We have proposed a qualitative framework to assess opinion formation models by systematically comparing their predictions with large-scale data sets taken from real-life surveys. First, we have introduced a *qualitative classification* of opinion distributions into five categories that account for increasing heterogeneity: *perfect consensus*, *consensus*, *polarization*, *clustering* and *dissensus*. Then, we have constructed *transition tables* to capture how an initial opinion distribution, belonging to a given category, can evolve over time into a final opinion distribution belonging to a possibly different category. The accuracy of an opinion formation model can be evaluated by comparing the *real transition table*, which displays the evolution between survey data taken in two subsequent occasions, with the *predicted transition table*, which displays the prediction generated by the model starting from initial survey data.

Our analysis of real opinion data from the World Values Survey^27–29^, shown in Fig. 3, provides insight into the evolution of real-life opinions, and in particular reveals that:

1. In real life, all possible transitions can occur.
2. The fraction of opinion distributions of each qualitative category appears to remain almost constant in each wave.
3. About half of the opinion distributions remain of the same category in subsequent waves.

Therefore, a fully realistic opinion formation model should be able to produce, with suitably chosen parameters, opinion distributions that recreate these three features. In particular, it should produce opinion distributions belonging to all the identified qualitative categories, starting not only from random initial conditions, but also from initial opinion distributions of each qualitative category.

Comparing real and predicted transition tables helps cast light onto the discrepancy between the evolution of real opinions and the predicted evolution generated by opinion formation models, thus identifying aspects of real-life opinion evolution that opinion formation models are not yet able to capture well, and suggesting directions to design improved and more realistic models. In particular, while the real transition tables are almost full matrices (Fig. 3), the predicted transition tables are typically sparse (Fig. 4): the models are inherently unable to yield some of the transitions observed in real data. Among the considered models, the Bounded Confidence model with intermediate confidence radius and the recently proposed Weighted-Median and Quantum Game models appear to be the most flexible, able to generate the richest variety of transitions and behaviors. However, there is still room for improvement.

The comparison between real and predicted transition tables highlights that improved opinion formation models should include flexible mechanisms able to both leave the opinion distribution category unchanged and produce any of the other distribution categories, under appropriate circumstances. We summarize some key observations, pointing at directions to improve existing models so as to match opinion transitions observed in real life.

1. Most models exhibit a strong agreement bias, resulting in an unrealistic tendency towards (perfect) consensus. This tendency could be mitigated by considering, e.g., the Friedkin-Johnsen model^66^, which takes into account not only individual self-confidences, but also individual susceptibilities to social influence; these additional parameters are however extremely challenging to estimate, especially in large-scale interaction networks.
2. There is no direct mechanism to produce dissensus, clustering or polarization starting from (perfect) consensus; however, these transitions do happen in real life. At the expense of the simplicity of the model, stochastic and random effects could be introduced through a noise component, representing the individuals’ free will and the unpredictability of their decisions^7^. The heterogeneity of the opinion distribution can also be increased by the presence of signed weights.
3. Most often, in the models the opinions change fast and significantly, typically converging towards an equilibrium state, whereas in real data there are plenty of examples of opinion distributions that remain *almost* constant and continue to change very slowly. This suggests that, as recently observed^67^, most of the actual social dynamics lead to transient, non-equilibrium phenomena: *ad-hoc* models should be developed to capture this effect. In this context, understanding the timescale of phenomena influencing opinion formation is crucial to map the time of model simulation to the time of real-world opinion evolution, a still unresolved challenge^67^.
4. Random initial opinion distributions are typically used when analyzing, or numerically simulating, opinion formation models. However, as shown in survey results^27–29^, this is not realistic: opinions tend to have characteristic qualitative distributions, which should be taken into account when evaluating opinion formation models. The critical role of initial conditions in opinion formation models has been pointed out as a long-overlooked problem^67^: different initial conditions can lead to grossly different final states and it is then fundamental to assign the initial opinion distribution appropriately, which remains an open challenge, in particular when large-scale interaction networks are considered.

We hope that the systematic evaluation tool we have provided, which can be used by researchers to gain insight into the features of real-life opinion evolution and to analyze existing or future opinion formation models, assessing their ability to reproduce real-life opinion data taken from any existing or future suitable dataset, can support the development of increasingly realistic opinion formation models. Other possible datasets that could be successfully used within the proposed framework are, for instance, the European Values Study^68^ and the Eurobarometer^69^.

## Methods

### Graphs and opinions

A directed graph (*digraph*) $\mathscr {G}$ with $N$ vertices can be represented by a set of vertices $\mathcal {V}=\{1, \dots , N\}$ and a set of weighted edges $\mathscr {E}\subseteq \{(i,j) :i,j \in \mathscr {V}\}$, where each edge (*i*, *j*) from vertex *j* to vertex *i* is associated with a real number (weight) $w_{ij}$. In the context of opinion dynamics, the vertices correspond to individuals, each associated with a time-varying opinion (individual *i* has opinion $x_i$) and $w_{ij}$ represents the *influence* that individual *j* exerts over individual *i*. In all the models we consider, the influence is positive (or zero, when no edge connects the two individuals) and all the influences exerted over an individual add up to a maximum value normalized to 1. Also, each individual is assumed to have self-confidence $w_{ii}>0$, for all $i\in \mathscr {V}$, which represents the persistence of belief in its own opinion. For our simulations, we generated strongly connected digraphs with small-world properties through the Watts-Strogatz algorithm^70^. For the French-DeGroot and Weighted-Median models the weights $w_{ij}$ were randomly generated.

### Opinion formation models

All the opinion formation models we consider evolve in discrete time, with opinions belonging to the set $[-1,1]$. They are agent based models, in which every individual or agent has a personal opinion. The opinion of agent *i* at time *k* is denoted as $x_i(k)$. Here we summarize the opinion update rules for the considered models: French-DeGroot, Weighted-Median, Bounded Confidence, and Quantum Game. These models were chosen because they are directly comparable, while for instance the Altafini model^20,71^ would require a *signed* graph, and the Friedkin-Johnsen model^66^ would require additional *susceptibility* coefficients. Also, the French-DeGroot and Bounded Confidence models are well known “benchmark” models that have been used as a basis for several extensions and refinements^72,73^.

The decision to evolve the models over small-world networks is backed up by a significant body of literature^74–76^ and is based on the fact that the two main features of small-world networks (high clustering coefficient and low average path distance) are typically observed in real-life interactions.

#### French-DeGroot model^2,12,13^

At every time step, the opinions of each individual are updated according to the rule:

$$\begin{aligned} x_i(t+1) = \sum _{j\in \mathscr {V}}w_{ij}x_j(t), \quad \forall i\in \mathscr {V}. \end{aligned}$$

#### Weighted-Median model^32^

At every time step, only the opinion of a single individual is updated. This individual is chosen randomly and moves to the opinion of another individual selected as follows:

$$\begin{aligned} x_i(t+1) = x^*, \end{aligned}$$

where $x^*\in \{x_1(t), \dots , x_N(t)\}$ is the opinion satisfying

$$\begin{aligned} \sum _{j:x_j(t)<x^*} w_{ij}\le \frac{1}{2}, \qquad \text {and} \qquad \sum _{j:x_j(t)>x^*}w_{ij}\le \frac{1}{2}. \end{aligned}$$

If more than one opinion satisfies these inequalities, then $x^*$ is taken as the opinion closest to $x_i(t)$.

#### Bounded confidence model^9^

This model is similar to the French-DeGroot model; however, at every time step, agent *i* is influenced by agent *j* if and only if $|x_i - x_j|\le r$, where *r* is the confidence radius. Mathematically the model evolves according the the following equation:

$$\begin{aligned} x_i(t+1) = |N_i|^{-1}\sum _{j\in N_i} x_j(t), \quad \forall i\in \mathscr {V}, \end{aligned}$$

where $N_i = \{j\in \mathcal {V}:|x_i(t)-x_j(t)|\le r\}$. We consider three versions of the Bounded Confidence model, each with a different confidence radius: $r=0.1$, $r=0.3$ and $r=0.7$.

#### Quantum game model^36^

In this model, at each time step, two randomly chosen agents interact pairwise. At each interaction, the agents have three options: *Keep* (keep their opinion), *Change* (take the other agent opinion), and *Agree* (take an intermediate opinion). The action each agent decides to take depends on the payoff. In this quantum model the payoff matrices depend on parameters *a*, *b*, *c* and the initial entangled state $|\psi _\text {in}\rangle$. For the simulations shown in the paper the values of the parameters were $a=1$, $b=3$, $c=1$, and $|\psi _\text {in}\rangle = \sqrt{1/2}|00\rangle + (1/2)|11\rangle + (1/2)|22\rangle$, as in one of the examples in the original paper^36^, so that the opinion formation law reduces to:

- If $d \le 0.25$, then the unique Nash Equilibrium is to *Agree*, hence the new opinion of both agents is the mean of their previous opinion.
- If $d > 0.25$, then the Nash Equilibrium with greatest payoff is to *Keep*, hence the new opinion of both agents is the same as their previous opinion;

where *d* is the opinion distance between agents. For more details, the reader is referred to the original work proposing the model^36^.

## Supplementary Information

Supplementary Information.

## Data Availability

The raw data used in this paper is freely available in the World Value Survey database^27–29^. The datasets generated from the raw data, from which the paper conclusions are drawn are available at https://giuliagiordano.dii.unitn.it/docs/papers/Framework_to_Analyze_Opinion_Formation_Models-Code.zip.

## Code availability

Simulation experiments, visualisation, and analysis were performed in MATLAB. The code necessary to perform the simulations that produce the results shown in the paper is available at https://giuliagiordano.dii.unitn.it/docs/papers/Framework_to_Analyze_Opinion_Formation_Models-Code.zip, together with the supplementary material that explains the code usage.
