# Supplementary material for: A framework to analyze opinion formation models
Source: Sci Rep. 2022 Aug 4;12:13441. doi: 10.1038/s41598-022-17348-z (PMC9352787; doi:10.1038/s41598-022-17348-z)
Supplement: Supplementary file 1 — Supplementary Information. [file 41598_2022_17348_MOESM1_ESM.pdf]

Supplementary Information: *A framework to analyze opinion formation models*

Carlos Andres Devia[1,*] and Giulia Giordano[1,2]

[1]Delft University of Technology, Delft Center for Systems and Control, Delft, 2628 CN, The Netherlands.
[2]University of Trento, Department of Industrial Engineering, Trento, 38123, Italy.
[*]c.a.deviapinzon@tudelft.nl

ABSTRACT

This document presents the Supplementary Information for the paper *A framework to analyze opinion formation models*. It includes a description of the simulation procedure followed to create the transition tables, of the model parameters, of the complete code, as well as the datasets and the generated tables. It also includes a step-by-step guide to use the provided code.

Contents

1 Simulations to produce Transition Tables **1**

2 Model Parameters **2**
- 2.1 French-DeGroot Model . . . 2
- 2.2 Weighted-Median Model . . . 3
- 2.3 Bounded Confidence Model . . . 3
- 2.4 Result and Analysis Significance . . . 3

3 Code usage **3**
- 3.1 Steps to reproduce the results . . . 3
- 3.2 Step by step instructions to replicate the results . . . 4
- 3.3 Data extraction from the World Values Survey results . . . 5
- 3.4 Small-World Network Creation . . . 5
- 3.5 Initial Opinion Creation . . . 6
- 3.6 Main Code . . . 6

4 Datasets **7**

5 Tables **9**

1 Simulations to produce Transition Tables

The World Values Survey results are assumed to be representative of the corresponding communities: for each country, question, and survey wave, if a different number of people in the same country were asked the same question at the same time, the resulting histogram would be a simple re-scaling of the original one (with the same 'shape'). Hence, not only we can re-scale histograms so as to evolve opinion formation models with more or less individuals than the real survey answers, but also we can think of the answers in different survey waves, for the same country, as given by the same people (which is not the case in reality). This allows us to compare predicted and real opinions.
The predicted transition table from Wave K to Wave $K+1$ is computed following these steps:

1. set the number of individuals for the model simulation, N;
2. take an initial histogram (corresponding to a particular country and question) from Wave K, then scale it so that the total sum of bin counts is equal to N and each bin count is an integer;

3. transform the scaled histogram into a set of N initial opinions: if bin k has n elements, then n individuals are assigned an initial opinion equal to the middle value of bin k;
4. generate the graph $\mathscr{G}$, over which the opinions evolve, as a strongly connected small-world network;
5. assign each initial opinion to a node in the graph;
6. evolve the opinions, with the given initial conditions, over the generated network graph, according to the update rule of the chosen opinion formation model, for T time steps;
7. classify the final opinions;
8. repeat steps 2 to 7 for every question and every country;
9. based on the qualitative classification of all the initial (*real*) and final (*predicted*) opinion distributions, construct the predicted transition table.

The above steps require four seemingly arbitrary choices that could change the outcome of the simulations: (i) the number N of considered individuals, (ii) the graph topology $\mathscr{G}$, (iii) the initial opinion assignment, and (iv) the number T of time steps for the model simulation.

The possible changes due to the number of individuals, the small-world network topology, and the initial opinion assignment were evaluated through an extensive simulation campaign. Models with $N = 100$, $N = 500$, and $N = 1000$ individuals were simulated. For each choice of N, 5 different small-world network topologies were generated, and for each network topology 5 different random initial opinion assignments were considered (thus, for each choice of N, 25 systems were evolved). For each network topology and random assignment of initial opinions, the transition table was computed, resulting in a total of 75 different transition tables (for each considered model). These transition tables were then averaged (entry by entry). For each entry, the variability is expressed as the difference between its maximum and minimum value across all 75 tables. The results are presented in Tables 10 and 11.

Concerning the number of time steps, the French-DeGroot and Bounded Confidence models were evolved for 50 iterations: since in these models every individual can change opinion at each time step, every individual had the opportunity to change opinion 50 times. In the Weighted Median model, only one individual changes opinion per each iteration, therefore for this model we considered 5000, 25000, and 50000 iterations for the graphs with 100, 500, and 1000 individuals respectively, so that *on average* each individual could change opinion 50 times and the results were comparable.

We allowed 50 opinion changes because of the type of questions, which involved opinions on values, and of the time interval between real survey waves, 5 years on average: opinions were then allowed to change at most 10 times per year. For more 'trivial' questions, or in a quickly changing opinion environment, more iterations could be allowed. It is important to stress that the results may vary depending on the total number of times an individual is allowed to change opinion: even a model known to always asymptotically lead to perfect consensus can produce a different distribution if it evolves for a short time. However, in our simulations we observed that the French-DeGroot model and the Bounded Confidence ($r = 0.7$) model reach consensus after few iterations, while the Bounded Confidence ($r = 0.1$) model leave the qualitative opinion distribution unchanged even over a very long simulation horizon, hence these models show a very small sensitivity with respect to the number of time steps.

2 Model Parameters

2.1 French-DeGroot Model

The weights w_{ij} were chosen from a uniform random distribution $U(0,1)$ and then normalized so that the corresponding adjacency matrix is row stochastic. Regardless of the chosen weights, if the graph is strongly connected, then the opinions (evolving according to the French-DeGroot model) will asymptotically converge to a common value, leading to perfect consensus.

However, it is possible to construct ad-hoc strongly connected networks so that the results can vary. One could for instance divide the vertices of the network in two groups and decrease the weights of edges between groups such that 'virtually' two strongly connected components are formed, despite the fact that they are technically one single strongly connected component. Under these circumstances, the opinions will first evolve towards polarization and then very slowly converge to perfect consensus. Thus, if one were to stop the simulation before the opinions have converged to consensus, the result will be interpreted as polarization.

Hence, the results presented in the paper will not hold for *any* possible digraph. However, they hold for most of the digraphs (and they hold *almost always* for randomly generated digraphs). Furthermore, the analysis and conclusions drawn from the results hold for any possible choice of the weights, namely: (*i*) there is a strong bias towards consensus not present in real life, and (*ii*) there is no mechanism to go from perfect consensus or consensus to polarization, clustering or disagreement.

2.2 Weighted-Median Model

As before, the weights w_{ij} were chosen from a uniform random distribution $U(0,1)$ and then normalized so that the corresponding adjacency matrix is row stochastic. The results presented in the paper can change if different weights are chosen: in fact, due to the stochastic nature of the model, even with the same initial conditions the results may vary. Furthermore, in this case there are no closed-form theoretical results that predict the asymptotic value of the opinions.

Despite the lack of theoretical results, looking at the foundations on which the model is built it is possible to conclude that it produces polarization, clustering, or dissensus with significantly low probability. The model is based on cognitive dissonance theory (in which agents tend to minimize their disagreement with their neighbours) and, as such, the opinions evolve with a strong bias towards agreement. Therefore, although the numerical results may vary with different digraphs, the conclusions and analysis are the same regardless of the choice of the weights.

2.3 Bounded Confidence Model

For this model the choice of weights w_{ij} depends on the digraph. If $N_{\hat{i}}$ is the set of in-neighbours of agent $\hat{i}$, then the weights $w_{\hat{i}\hat{j}}$ are the same for all $\hat{j} \in N_{\hat{i}}$ and equal to $|N_{\hat{i}}|^{-1}$. This is done to guarantee that the corresponding adjacency matrix is row stochastic at every time step.

Therefore, the only parameter of the model is the confidence radius. In principle, every agent could have different confidence radius, and even an "asymmetric" confidence radius depending on whether the opinion of the other is larger or smaller. So, the results presented in the paper can change if different weights are chosen, but not significantly. At its core, the Bounded Confidence model can be seen as the evolution of subgroups of agents (the "strongly connected components", depending on the agents opinions and their confidence radius, whose composition evolves over time depending on how the opinions change), whose opinions evolve simultaneously according to the French-DeGroot model. Therefore, the number of final opinions is the same as the number of final strongly connected components. The smaller the confidence radius, the larger the number of different final opinions. Of course, if the confidence radius is too small, then the opinions remain essentially unchanged, as shown by model BC1.

Hence, unlike the French-DeGroot model, for some choice of the parameters the Bounded Confidence model can produce polarization, clustering, and dissensus; however, these outcomes result from dividing the population in small groups, each converging to a common opinion. It is not an active separation of opinions, but a passive division. Like the French-DeGroot model, this model also lacks mechanisms to produce polarization, clustering, and dissensus starting from consensus and perfect consensus. Furthermore, the absence of consensus for a non-trivial confidence radius evidences the bias towards perfect consensus that is inherited from the dynamics of the French-DeGroot model.

2.4 Result and Analysis Significance

For different choices of the parameters (weights or confidence radius), the results presented in Tables 10 and 11 may be slightly different. Nevertheless, the analysis and conclusions drawn from these tables will be conceptually the same. Although the models could produce polarization, clustering, and dissensus for some particular networks (chosen ad-hoc) and for short enough evolution times, still the models lack the mechanisms that would allow them to produce such outcomes starting from consensus or perfect consensus. Furthermore, their inherent bias towards perfect consensus shapes the dynamic opinion evolution regardless of the chosen parameters.

3 Code usage

3.1 Steps to reproduce the results

All simulations were performed using MATLAB. To obtain the presented results, three steps must be followed:

1. Prepare auxiliary files.
2. Execute the main code.
3. Interpret the results.

Steps:

1. Prepare auxiliary files:
   (a) Download and suitably format the survey results from the World Values Survey Data. This includes:
      i. Determine the question number of each wave corresponding to the desired questions.

ii. Download the .R files.
iii. Using R, convert the .R files to .csv.
iv. Read the .csv files in MATLAB and extract the survey answers to the desired questions.
v. Determine all the countries that have answered the desired questions for Waves 5, 6, and 7.
vi. Extract and store the survey answers for each wave, question, and country in cells to be used later.

(b) Create 5 different small-world networks to be the initial digraphs. This is done for networks with 100, 500, and 1000 vertices. The digraphs are strongly connected and are constructed based on the Watts-Strogatz Small-World Graph Model (for more details, see the corresponding subsection).

(c) Using the real answers, generate the scaled initial opinions for 100, 500, and 1000 agents (for more details, see the corresponding subsection).

2. Execute the main code: This simply consists of the execution of the `DataProcessing_Wx_My.m` files, where x is the wave index (1 or 2) and y is the method index (1 to 5). Each .m file will create a .mat file called `Tables_wave_x_method_y.mat` (where x and y are as before) which contains the main results.

3. Interpret the results: Execute the `ResultsScript.m`, which will generate .tex files including the transition tables.

3.2 Step by step instructions to replicate the results

Important: The threshold value denoted by T_2 in the paper is called `alpha` in all the MATLAB scripts. Also, due to the random shuffling of initial conditions, the results may be slightly different even when starting with the same initial opinions and initial network.

1. Go to: `https://www.worldvaluessurvey.org/WVSDocumentationWV5.jsp`.

2. Click on: `WV5 Data R v20180912` to download the file `F00007944-WV5_Data_R_v20180912.rds`.

3. Open the downloaded file in R.

4. Type `filename <- file.choose()` and select the file.

5. Type `Wave5Table <- readRDS(filename)`.

6. Type `write.csv(Wave5Table,“Wave_5.csv”` to save the table as csv.

7. Go to: `https://www.worldvaluessurvey.org/WVSDocumentationWV6.jsp`.

8. Click on: `WV6 Data R v20201117.zip` to download the file `WV6_Data_R_v20201117.rdata`.

9. In R, type `write.csv(WV6_Data_R_v20201117, “Wave_6.csv”` to save the table as csv.

10. Go to: `https://www.worldvaluessurvey.org/WVSDocumentationWV7.jsp`.

11. Click on `WVS Cross-National Wave 7 csv v2 0.zip` to get the file `WVS_Cross-National_Wave_7_csv_v2_0`

12. Rename the file to `Wave_7.csv`.

13. Run the MATLAB script `RealHistAnalysis.m`. This script will read the .csv files and return a .mat file called `RealDataHist.mat`, among other things, this file will contain the answers to the questions that were answered in Waves 5, 6, and 7 for every country that answered all waves.

14. Run the MATLAB script `InitialConditionCreation.m`. This script will create the initial networks and initial opinions by executing the scripts `InitialNetwork.m` and `InitialOpinions.m` respectively.

15. Run the `DataProcessing_Wx_My.m` scripts for x=1,2 and y=1,2,3,4,5. These scripts evolve each of the initial conditions for Wave 5 (x=1) and Wave 6 (x=2), for each one of the considered opinion formation models (y=1, ..., 5). These scripts are identical, except for y=2 where the *total time* variable is different, because model 2 is the Weighted-Median model, where only one individual updates its opinion at each time, therefore more time steps are needed for each individual to update on average 50 times. The output of each of these scripts is the .mat file `Tables_wave_x_method_y.mat`, which contains the 75 predicted transition tables for Wave x and method y.

16. Run `ResultsScript.m`. This script takes the real data from `RealDataHist.mat` to create the real transition tables and also the predicted data from `Tables_wave_x_method_y.mat` to compute the average predicted transition tables that appear in the manuscript. Finally, it creates a .tex file that displays the tables.

Note: by executing steps from 1 to 16, the results obtained may differ from the ones presented in the paper. If the networks and initial conditions are different, then there may also be some differences in the results. This does not affect the conclusions drawn from the paper. Moreover, it is possible to replicate the results obtained in the paper if steps 1 to 13 are skipped and instead the initial conditions provided in the datasets 8 to 25 are used. By doing so, the final results will be the same as those reported in the paper, which are also in the datasets 26 to 35.

3.3 Data extraction from the World Values Survey results

The first step is to obtain the survey answers from the World Values Survey results. To do this first it was necessary to find the ID of all the desired questions for each wave (See Tables 13, 14, and 15). Then execute the script `RealHistAnalysis.m` which is explained in Algorithm 1.

Algorithm 1 `RealHistAnalysis.m`

Read the .csv for waves 5, 6, and 7.
Obtain all the different nationalities for each wave. Call C the set of all these countries.
Determine which countries have answers in all waves.
for Answers to wave 5, 6, and 7 **do**
    for For question $a \in \{1,\dots,25\}$ **do**
        for For country $b \in C$ **do**
            Select the answers to question a given in country b.
            Save the answers in a cell
        end for
    end for
end for
Save all the answers divided by wave, question, and country in the .mat file `RealDataHist.mat`

3.4 Small-World Network Creation

The initial networks were generated by the `InitialNetwork.m` script, detailed in Algorithm 2. It takes only the number of vertices the network has N.

Algorithm 2 `InitialNetwork.m` script

Require: N
Determine randomly the number of connections C, rewiring probability coefficient γ, and bidirectional probability coefficient γ
Execute the `SmallWorldNetwork.m` script to create a Small-World directed network.
Assign positive weights from a uniform distribution to the directed network.
Normalize the weights such that the corresponding adjacency matrix is row stochastic.

The Small-World Network was created based on the Watts–Strogatz model, using Algorithm 3. It takes the parameters N, number of agents; C, number of connections; β, rewiring probability coefficient; and γ, bidirectional probability coefficient.

Algorithm 3 `SmallWorldNetwork.m` script

Require: N, C, β, γ
Create a ring directed graph with N vertices and C connections: arrange the N vertices in a circle and connect vertex i with itself and the next $C - i$ vertices clock-wise.
Rewire the target vertex of each edge with probability β, except if the edge is a self-loop.
Make each edge bidirectional with probability γ. If the edge is uni-directional set the direction randomly (equal probability for each direction)

3.5 Initial Opinion Creation

Given a set of K answers to a Likert-10 scale question, denoted X (i.e. $X \in \{1,2,\dots,10\}^K$). This set is transformed into initial opinions for N agents using Algorithm 4. The set of final opinions is denoted $\tilde{X}$.

Algorithm 4 `InitialOpinions.m` script

Require: N, X

1: Compute the type of opinion distribution that the histogram of X is. Denote this type by *InitialType*.
2: For $k = 1,\dots,10$, compute size of bin k, denoted $H(k)$, as the number of answers in X that are equal to k. Clearly $\sum_{k=1,\dots,10} H(k) = K$.
3: Normalize the bins by a factor N/K. To avoid fraction numbers take the floor function. Rename these new bins $\hat{H}(k)$. Mathematically it follows that $\hat{H}(k) = \lfloor \frac{N}{K} H(k) \rfloor$.
4: Compute the number of opinions that are unassigned $\xi = K - \sum_{k=1,\dots,10} \hat{H}(k)$
5: Assign the remaining ξ opinions uniformly randomly to the bins $\hat{H}(k)$. Denote the final bins $\tilde{H}(k)$. At this point $\sum_{k=1,\dots,10} \tilde{H}(k) = N$.
6: Create an empty vector $\tilde{X}$ of N coefficients.
7: Divide the interval $[-1,1]$ in 10 equal subintervals. Denote by $\sigma(k)$ the value at the middle of subinterval k.
8: For bin $k \in \{1,2,\dots,N\}$ add $\tilde{H}(k)$ opinions with value $\sigma(k)$ to the opinion vector $\tilde{K}$.
9: Compute the type of opinion distribution that the histogram of $\tilde{X}$ is. Denote this type by *InitialType2*.
10: If *InitialType* and *InitialType2* are different then repeat steps 3 to 9 (due to the randomness in the assignment of the remaining opinions in step 5 it may happen that after repeating the process the initial and final opinion distributions belong to the same type). If the types keeps being different, then at step 3 normalize by $0.7(N/K)$ (this increases the possibility of belonging to the same type).
11: Shuffle randomly the opinions in $\tilde{X}$.

The result of executing Algorithm 4 is an opinion distribution $\tilde{X}$ with N agents that is of the same type as the original set of answers X and that in addition has values between -1 and 1.

Algorithm 4 is executed in each of the 2229 sets of survey answers 3 times, for $N = 100$, $N = 500$, and $N = 1000$. Denote my $X_{a,b}(N)$ the set of answers with N elements to question $a \in \{1,\dots,30\}$ in country $b \in \{1,\dots,25\}$. And by $X(N)$ the set of all opinion distributions $X(N) = \{X_{a,b}(N) \mid a \in \{1,\dots,30\}\ b \in \{1,\dots,25\}\}$.

3.6 Main Code

The script `DataProcessing_Wx_My.m` is in charge of computing the 75 predicted transition tables from wave 5 to 6 (if x=1) or wave 6 to 7 (if x=2) for method y (FG if y=1, MW if y=2, BC1 if y=3, BC2 if y=4, and BC3 if y=5). The functioning of this script is presented in Algorithm 5

Algorithm 5 `DataProcessing_Wx_My.m` script

Require: N, C, β, γ

Create a 5×5 matrix with zeros. This will be the predicted transition matrix. Denote it M.
for Initial opinions with $N = 100$, $N = 500$, and $N = 1000$ **do**
  for The initial small-world network $\mathcal{G}_j$ with N vertices, where $j = 1,2,3,4,5$ **do**
    for 5 repetitions **do**
      for Question $a = 1,\dots,30$ **do**
        for Country $b = 1,\dots,25$ **do**
          Shuffle randomly the initial opinions.
        end for
      end for
      Execute the `PredictedTransitionTable.m` script to compute the predicted transition table with the shuffled initial conditions (See Algorithm 6).
      Store the predicted transition table.
    end for
  end for
end for

Algorithm 6 `PredictedTransitionTable.m` script

```
for Question a = 1,...,30 do
    for Country b = 1,...,25 do
        Assign initial opinion i to agent i in the digraph for i = 1,...,N.
        Evolve the agent's opinions according to the selected method (y) for T time steps (T = 50 if y=1, 3, 4, and 5. If y=2, then T = 5000 for N = 100; T = 25000 for N = 500; and T = 50000 for N = 1000)
        Compute the initial opinion distribution type InitialType.
        Compute the final opinion distribution type FinalType.
    end for
end for
for Question a = 1,...,30 do
    for Country b = 1,...,25 do
        Increase the counter in row InitialType and column FinalType in the predicted transition matrix M.
    end for
end forreturn The predicted transition table.
```

4 Datasets

These are all the data and code used to obtain the results in the paper.

- `F00007944-WV5_Data_R_v20180912.rds`: Raw data for the survey results in Wave 5 obtained from the World Values Survey.
- `WV6_Data_R_v20201117.rdata`: Raw data for the survey results in Wave 6 obtained from the World Values Survey.
- `WVS_Cross-National_Wave_7_csv_v2_0.csv`: Raw data for the survey results in Wave 7 obtained from the World Values Survey.
- `Wave_5.csv`: Wave 5 results used by the script `RealHistAnalysis.m` to produce the data in `RealDataHist.mat`.
- `Wave_6.csv`: Wave 6 results used by the script `RealHistAnalysis.m` to produce the data in `RealDataHist.mat`.
- `Wave_7.csv`: Wave 7 results used by the script `RealHistAnalysis.m` to produce the data in `RealDataHist.mat`.
- `RealDataHist.mat`: Data processed from the World Value Survey created by the `RealHistAnalysis.m` script.
- `InitialNetwork_1_100_agents.mat`: Initial network number 1 with 100 agents.
- `InitialNetwork_1_500_agents.mat`: Initial network number 1 with 500 agents.
- `InitialNetwork_1_1000_agents.mat`: Initial network number 1 with 1000 agents.
- `InitialNetwork_2_100_agents.mat`: Initial network number 2 with 100 agents.
- `InitialNetwork_2_500_agents.mat`: Initial network number 2 with 500 agents.
- `InitialNetwork_2_1000_agents.mat`: Initial network number 2 with 1000 agents.
- `InitialNetwork_3_100_agents.mat`: Initial network number 3 with 100 agents.
- `InitialNetwork_3_500_agents.mat`: Initial network number 3 with 500 agents.
- `InitialNetwork_3_1000_agents.mat`: Initial network number 3 with 1000 agents.
- `InitialNetwork_4_100_agents.mat`: Initial network number 4 with 100 agents.
- `InitialNetwork_4_500_agents.mat`: Initial network number 4 with 500 agents.
- `InitialNetwork_4_1000_agents.mat`: Initial network number 4 with 1000 agents.
- `InitialNetwork_5_100_agents.mat`: Initial network number 5 with 100 agents.

- `InitialNetwork_5_500_agents.mat`: Initial network number 5 with 500 agents.
- `InitialNetwork_5_1000_agents.mat`: Initial network number 5 with 1000 agents.
- `InitialOpinions_100_agents.mat`: Initial opinions with 100 agents.
- `InitialOpinions_500_agents.mat`: Initial opinions with 500 agents.
- `InitialOpinions_1000_agents.mat`: Initial opinions with 1000 agents.
- `Tables_wave_1_method_1.mat`: The 75 predicted transition tables obtained with method 1 (French-DeGroot) from wave 5 to wave 6.
- `Tables_wave_1_method_2.mat`: The 75 predicted transition tables obtained with method 2 (Weighted-Median) from wave 5 to wave 6.
- `Tables_wave_1_method_3.mat`: The 75 predicted transition tables obtained with method 3 (Bounded Confidence with confidence radius 0.1) from wave 5 to wave 6.
- `Tables_wave_1_method_4.mat`: The 75 predicted transition tables obtained with method 3 (Bounded Confidence with confidence radius 0.3) from wave 5 to wave 6.
- `Tables_wave_1_method_5.mat`: The 75 predicted transition tables obtained with method 3 (Bounded Confidence with confidence radius 0.7) from wave 5 to wave 6.
- `Tables_wave_2_method_1.mat`: The 75 predicted transition tables obtained with method 1 (French-DeGroot) from wave 6 to wave 7.
- `Tables_wave_2_method_2.mat`: The 75 predicted transition tables obtained with method 2 (Weighted-Median) from wave 6 to wave 7.
- `Tables_wave_2_method_3.mat`: The 75 predicted transition tables obtained with method 3 (Bounded Confidence with confidence radius 0.1) from wave 6 to wave 7.
- `Tables_wave_2_method_4.mat`: The 75 predicted transition tables obtained with method 3 (Bounded Confidence with confidence radius 0.3) from wave 6 to wave 7.
- `Tables_wave_2_method_5.mat`: The 75 predicted transition tables obtained with method 3 (Bounded Confidence with confidence radius 0.7) from wave 6 to wave 7.

5 Tables

Table 1. EXAMPLE: Transition table from Wave 5 to Wave 6.

		Wave 6 (predicted)				
		P.C.	Co	Po	Cl	Di
Wave 5	P.C.	2	0	0	0	0
	Co	0	0	0	0	0
	Po	1	0	0	0	0
	Cl	0	0	0	1	0
	Di	0	0	0	0	0

Table 2. EXAMPLE: Transition table from Wave 5 to Wave 6.

		Wave 6 (real)				
		P.C.	Co	Po	Cl	Di
Wave 5	P.C.	0	0	1	1	0
	Co	0	0	0	0	0
	Po	0	0	1	0	0
	Cl	0	0	0	0	1
	Di	0	0	0	0	0

Table 3. Classification of all real data histograms in perfect consensus (P.C), consensus (Co), polarization (Po), clustering (Cl), and dissensus (Di) for wave 5.

P.C.	178
Co	86
Po	37
Cl	128
Di	246

Table 4. Classification of all real data histograms in perfect consensus (P.C), consensus (Co), polarization (Po), clustering (Cl), and dissensus (Di) for wave 6.

P.C.	155
Co	98
Po	34
Cl	133
Di	255

Table 5. Classification of all real data histograms in perfect consensus (P.C), consensus (Co), polarization (Po), clustering (Cl), and dissensus (Di) for wave 7.

P.C.	151
Co	74
Po	41
Cl	150
Di	259

Table 6. Transition table from Wave 5 to Wave 6.

		Wave 6				
		P.C.	Co	Po	Cl	Di
Wave 5	P.C.	130	23	6	2	17
	Co	12	32	0	13	29
	Po	2	1	12	7	15
	Cl	3	14	5	53	53
	Di	8	28	11	58	141

Table 7. Transition table from Wave 6 to Wave 7.

		Wave 7				
		P.C.	Co	Po	Cl	Di
Wave 6	P.C.	129	10	5	5	6
	Co	11	27	1	18	41
	Po	2	0	16	9	7
	Cl	3	11	7	58	54
	Di	6	26	12	60	151

Table 8. Average transition table from Wave 5 to Wave 6.Each cell contains the average of the 75 values in the corresponding cell for each transition table. The dispersion of these values is measured by the difference between the maximum and minimum in the corresponding cell across all 75 tables, and it is represented by the shade of blue. A white cell means zero difference, a cell with color represents a variation of half the maximum variation for that method, and a cell with color represents the maximum variation for that method. The maximum difference for each method is: FG 18, WM 225, BC1 0, BC2 7, BC3 0, and QG 120.

Method	Wave 5	P.C.	Co	Po	Cl	Di
FG	P.C.	177.69	0.31	0	0	0
	Co	85.84	0.15	0	0	0.01
	Po	36.72	0.28	0	0	0
	Cl	127.31	0.68	0	0	0.01
	Di	245.01	0.97	0	0	0.01
WM	P.C.	177.73	0.27	0	0	0
	Co	81.29	4.05	0	0.12	0.53
	Po	34.65	0.51	0.04	1.16	0.64
	Cl	119.77	3.69	0.07	1.25	3.21
	Di	231.48	8.55	0.01	1.73	4.23
BC1	P.C.	178	0	0	0	0
	Co	0	86	0	0	0
	Po	0	0	37	0	0
	Cl	0	0	0	128	0
	Di	0	0	0	0	246
BC2	P.C.	178	0	0	0	0
	Co	86	0	0	0	0
	Po	26.67	0	1.67	8.67	0
	Cl	90.33	0	0.33	37.33	0
	Di	186.67	0	1.33	58	0
BC3	P.C.	178	0	0	0	0
	Co	86	0	0	0	0
	Po	37	0	0	0	0
	Cl	128	0	0	0	0
	Di	246	0	0	0	0
QG	P.C.	178	0	0	0	0
	Co	79.33	0.13	0.15	6.37	0.01
	Po	16.25	0	10.2	10.55	0
	Cl	30.39	0.03	3.23	94.35	0.01
	Di	94.47	1.44	10.31	139.52	0.27

(Columns: Wave 6)

Table 9. Average transition table from Wave 6 to Wave 7.Each cell contains the average of the 75 values in the corresponding cell for each transition table. The dispersion of these values is measured by the difference between the maximum and minimum in the corresponding cell across all 75 tables, and it is represented by the shade of blue. A white cell means zero difference, a cell with color [light blue] represents a variation of half the maximum variation for that method, and a cell with color [blue] represents the maximum variation for that method. The maximum difference for each method is: FG 18, WM 225, BC1 0, BC2 7, BC3 0, and QG 120.

Method	Wave 6 \ Wave 7	P.C.	Co	Po	Cl	Di
FG	P.C.	154.84	0.16	0	0	0
	Co	97.83	0.17	0	0	0
	Po	33.8	0.19	0	0	0.01
	Cl	132.57	0.43	0	0	0
	Di	254.15	0.84	0	0	0.01
WM	P.C.	154.59	0.4	0	0	0.01
	Co	92.59	4.52	0	0.07	0.83
	Po	31.92	0.4	0.15	1.05	0.48
	Cl	124.61	3.96	0.12	1.27	3.04
	Di	240.13	8.85	0.03	1.51	4.48
BC1	P.C.	155	0	0	0	0
	Co	0	98	0	0	0
	Po	0	0	34	0	0
	Cl	0	0	0	133	0
	Di	0	0	0	0	255
BC2	P.C.	155	0	0	0	0
	Co	98	0	0	0	0
	Po	20.33	0	4.33	9.33	0
	Cl	92.33	0	2.33	38.33	0
	Di	210	0	0	45	0
BC3	P.C.	155	0	0	0	0
	Co	98	0	0	0	0
	Po	34	0	0	0	0
	Cl	133	0	0	0	0
	Di	255	0	0	0	0
QG	P.C.	154.99	0.01	0	0	0
	Co	70.56	11.95	0.49	12.71	2.29
	Po	11.6	0	15.83	6.55	0.03
	Cl	19.41	0.57	3.89	105.11	4.01
	Di	66.69	10.29	14.79	127.81	35.41

Table 10. Difference between the maximum and minimum value for all the possible transitions in the 6 models, i.e. for all cells in the 5 transition tables across all the 75 transition tables from Wave 5 to Wave 6.

FG	Wave 5 \ Wave 6	P.C.	Co	Po	Cl	Di
	P.C.	7	7	0	0	0
	Co	4	3	0	0	1
	Po	6	6	0	0	0
	Cl	16	16	0	0	1
	Di	18	18	0	0	1

WM	Wave 5 \ Wave 6	P.C.	Co	Po	Cl	Di
	P.C.	7	7	0	0	0
	Co	73	65	0	4	11
	Po	36	9	1	23	12
	Cl	125	57	2	20	53
	Di	224	137	1	28	69

BC1	Wave 5 \ Wave 6	P.C.	Co	Po	Cl	Di
	P.C.	0	0	0	0	0
	Co	0	0	0	0	0
	Po	0	0	0	0	0
	Cl	0	0	0	0	0
	Di	0	0	0	0	0

BC2	Wave 5 \ Wave 6	P.C.	Co	Po	Cl	Di
	P.C.	0	0	0	0	0
	Co	0	0	0	0	0
	Po	2	0	1	1	0
	Cl	2	0	1	3	0
	Di	7	0	1	6	0

BC3	Wave 5 \ Wave 6	P.C.	Co	Po	Cl	Di
	P.C.	0	0	0	0	0
	Co	0	0	0	0	0
	Po	0	0	0	0	0
	Cl	0	0	0	0	0
	Di	0	0	0	0	0

QG	Wave 5 \ Wave 6	P.C.	Co	Po	Cl	Di
	P.C.	0	0	0	0	0
	Co	8	2	2	8	1
	Po	4	0	12	10	0
	Cl	12	1	7	15	1
	Di	30	4	13	31	2

Table 11. Difference between the maximum and minimum value for all the possible transitions in the 6 models, i.e. for all cells in the 5 transition tables across all the 75 transition tables from Wave 6 to Wave 7.

Model	Wave 6 \ Wave 7	P.C.	Co	Po	Cl	Di
FG	P.C.	3	3	0	0	0
	Co	6	6	0	0	0
	Po	7	6	0	0	1
	Cl	8	8	0	0	0
	Di	18	17	0	0	1
WM	P.C.	8	8	0	0	1
	Co	85	78	0	2	15
	Po	32	8	4	18	9
	Cl	129	65	3	23	53
	Di	225	135	2	25	73
BC1	P.C.	0	0	0	0	0
	Co	0	0	0	0	0
	Po	0	0	0	0	0
	Cl	0	0	0	0	0
	Di	0	0	0	0	0
BC2	P.C.	0	0	0	0	0
	Co	0	0	0	0	0
	Po	4	0	2	2	0
	Cl	3	0	2	5	0
	Di	2	0	0	2	0
BC3	P.C.	0	0	0	0	0
	Co	0	0	0	0	0
	Po	0	0	0	0	0
	Cl	0	0	0	0	0
	Di	0	0	0	0	0
QG	P.C.	1	1	0	0	0
	Co	59	39	4	18	11
	Po	6	0	17	13	1
	Cl	32	4	7	21	17
	Di	88	37	19	97	120

Table 12. Countries considered in our data analysis

Country ID in our code	Country code in the WVS dataset	Name of the country
1	32	Argentina
2	36	Australia
3	76	Brazil
4	152	Chile
5	156	China
6	170	Colombia
7	196	Cyprus
8	818	Egypt
9	276	Germany
10	344	Hong kong
11	368	Iraq
12	400	Jordan
13	392	Japan
14	458	Malaysia
15	484	Mexico
16	554	New Zealand
17	604	Peru
18	642	Romania
19	643	Russia
20	410	South Korea
21	764	Thailand
22	158	Taiwan
23	792	Turkey
24	804	Ukraine
25	840	United States

Table 13. Questions considered in our analysis (part 1 of 3)

Question ID	Number in the questionnaire of			Question (all answers are given in a scale from 1 to 10)
	Wave 5	Wave 6	Wave 7	
1	46	55	48	Some people feel they have completely free choice and control over their lives, while other people feel that what they do has no real effect on what happens to them. Please use this scale where 1 means **no choice at all** and 10 means **a great deal of choice** to indicate how much freedom of choice and control you feel you have over the way your life turns out
2	22	23	49	All things considered, how satisfied are you with your life as a whole these days? Using this card on which 1 means you are **completely dissatisfied** and 10 means you are **completely satisfied** where would you put your satisfaction with your life as a whole?
3	68	59	50	How satisfied are you with the financial situation of your household? Please use this card again to help with your answer (1 is completely dissatisfied, 10 is completely satisfied)
4	116	96	106	How would you place your views on this scale? 1 means you completely agree with the statement **Incomes should be made more equal**; 10 means you completely agree with the statement **We need larger income differences as incentives for individual effort**. And if your views fall somewhere in between, you can choose any number in between.
5	117	97	107	How would you place your views on this scale? 1 means you completely agree with the statement **Private ownership of business and industry should be increased**; 10 means you completely agree with the statement **Government ownership of business and industry should be increased**. And if your views fall somewhere in between, you can choose any number in between.
6	118	98	108	How would you place your views on this scale? 1 means you completely agree with the statement **The government should take more responsibility to ensure that everyone is provided for**; 10 means you completely agree with the statement **People should take more responsibility to provide for themselves**. And if your views fall somewhere in between, you can choose any number in between.
7	119	99	109	How would you place your views on this scale? 1 means you completely agree with the statement **Competition is good. It stimulates people to work hard and develop new ideas**; 10 means you completely agree with the statement **Competition is harmful. It brings out the worst in people**. And if your views fall somewhere in between, you can choose any number in between.
8	120	100	110	How would you place your views on this scale? 1 means you completely agree with the statement **In the long run, hard work usually brings a better life**; 10 means you completely agree with the statement **Hard work doesn't generally bring success—it's more a matter of luck and connections**. And if your views fall somewhere in between, you can choose any number in between.
9	91	192	158	How much you agree or disagree with the statement **Science and technology are making our lives healthier, easier, and more comfortable.**. For this questions, a 1 means that you "completely disagree" and a 10 means that you "completely agree."

Table 14. Questions considered in our analysis (part 2 of 3)

Question ID	Number in the questionnaire of			Question (all answers are given in a scale from 1 to 10)
	Wave 5	Wave 6	Wave 7	
10	92	193	159	How much you agree or disagree with the statement **Because of science and technology, there will be more opportunities for the next generation.**. For this questions, a 1 means that you "completely disagree" and a 10 means that you "completely agree."
11	94	194	160	How much you agree or disagree with the statement **We depend too much on science and not enough on faith.**. For this questions, a 1 means that you "completely disagree" and a 10 means that you "completely agree."
12	123	197	163	All things considered, would you say that the world is better off, or worse off, because of science and technology? 1 means that "the world is a lot worse off," and 10 means that "the world is a lot better off."
13	192	152	164	How important is God in your life? 10 means "very important" and 1 means "not at all important."
14	198	198	177	Indicate if the action of **Claiming government benefits to which you are not entitled** can be never justified (1); always justified (10); or something in between in a scale from 1 to 10.
15	200	201	180	Indicate if the action of **Cheating on taxes if you have a chance** can be never justified (1); always justified (10); or something in between in a scale from 1 to 10.
16	201	202	181	Indicate if the action of **Someone accepting a bribe in the course of their duties** can be never justified (1); always justified (10); or something in between in a scale from 1 to 10.
17	202	203	182	Indicate if the action of **Homosexuality** can be never justified (1); always justified (10); or something in between in a scale from 1 to 10.
18	204	204	184	Indicate if the action of **Abortion** can be never justified (1); always justified (10); or something in between in a scale from 1 to 10.
19	205	205	185	Indicate if the action of **Divorce** can be never justified (1); always justified (10); or something in between in a scale from 1 to 10.
20	207	207	187	Indicate if the action of **Suicide** can be never justified (1); always justified (10); or something in between in a scale from 1 to 10.
21	208	208	189	Indicate if the action of **For a man to beat his wife** can be never justified (1); always justified (10); or something in between in a scale from 1 to 10.
22	152	131	241	Is **Governments tax the rich and subsidize the poor.** an essential characteristic of democracy? Use this scale where 1 means "not at all an essential characteristic of democracy" and 10 means it definitely is "an essential characteristic of democracy"
23	153	132	242	Is **Religious authorities interpret the laws.** an essential characteristic of democracy? Use this scale where 1 means "not at all an essential characteristic of democracy" and 10 means it definitely is "an essential characteristic of democracy"
24	154	133	243	Is **People choose their leaders in free elections.** an essential characteristic of democracy? Use this scale where 1 means "not at all an essential characteristic of democracy" and 10 means it definitely is "an essential characteristic of democracy"

Table 15. Questions considered in our analysis (part 3 of 3)

Question ID	Number in the questionnaire of			Question (all answers are given in a scale from 1 to 10)
	Wave 5	Wave 6	Wave 7	
25	155	134	244	Is **People receive state aid for unemployment.** an essential characteristic of democracy? Use this scale where 1 means “not at all an essential characteristic of democracy” and 10 means it definitely is “an essential characteristic of democracy”
26	156	135	245	Is **The army takes over when government is incompetent.** an essential characteristic of democracy? Use this scale where 1 means “not at all an essential characteristic of democracy” and 10 means it definitely is “an essential characteristic of democracy”
27	157	136	246	Is **Civil rights protect people’s liberty against oppression.** an essential characteristic of democracy? Use this scale where 1 means “not at all an essential characteristic of democracy” and 10 means it definitely is “an essential characteristic of democracy”
28	161	139	249	Is **Women have the same rights as men.** an essential characteristic of democracy? Use this scale where 1 means “not at all an essential characteristic of democracy” and 10 means it definitely is “an essential characteristic of democracy”
29	162	140	250	How important is it for you to live in a country that is governed democratically? On this scale where 1 means it is “not at all important” and 10 means “absolutely important" what position would you choose?
30	163	141	251	And how democratically is this country being governed today? Again using a scale from 1 to 10, where 1 means that it is “not at all democratic” and 10 means that it is “completely democratic,” what position would you choose?
